# Controlled potential electro-oxidation of genomic DNA

**DOI:** 10.1371/journal.pone.0190907

**Published:** 2018-01-11

**Authors:** Vytas Reipa, Donald H. Atha, Sanem H. Coskun, Christopher M. Sims, Bryant C. Nelson

**Affiliations:** Materials Measurement Laboratory, Biosystems and Biomaterials Division, National Institute of Standards and Technology, Gaithersburg, Maryland, United States of America; US Naval Research Laboratory, UNITED STATES

## Abstract

Exposure of mammalian cells to oxidative stress can result in DNA damage that adversely affects many cell processes. Lack of dependable DNA damage reference materials and standardized measurement methods, despite many case-control studies hampers the wider recognition of the link between oxidatively degraded DNA and disease risk. We used bulk electrolysis in an electrochemical system and gas chromatographic mass spectrometric analysis (GC/MS/MS) to control and measure, respectively, the effect of electrochemically produced reactive oxygen species on calf thymus DNA (ct-DNA). DNA was electro-oxidized for 1 h at four fixed oxidizing potentials (E = 0.5 V, 1.0 V, 1.5 V and 2 V (vs Ag/AgCl)) using a high surface area boron-doped diamond (BDD) working electrode (WE) and the resulting DNA damage in the form of oxidatively-modified DNA lesions was measured using GC/MS/MS. We have shown that there are two distinct base lesion formation modes in the explored electrode potential range, corresponding to 0.5 V < E < 1.5 V and E > 1.5 V. Amounts of all four purine lesions were close to a negative control levels up to E = 1.5 V with evidence suggesting higher levels at the lowest potential of this range (E = 0.5 V). A rapid increase in all base lesion yields was measured when ct-DNA was exposed at E = 2 V, the potential at which hydroxyl radicals were efficiently produced by the BDD electrode. The present results demonstrate that controlled potential preparative electrooxidation of double-stranded DNA can be used to purposely increase the levels of oxidatively modified DNA lesions in discrete samples. It is envisioned that these DNA samples may potentially serve as analytical control or quality assurance reference materials for the determination of oxidatively induced DNA damage.

## Introduction

Exogeneous disturbances (exposure to ionizing radiation or chemical toxicants) or endogenous perturbations (inflammation or elevated iron content) in the normal redox status of cells can produce oxidizing species that can potentially damage cellular components (e.g., proteins, lipids, and DNA) [1]. These oxidizing species, generally known as reactive oxygen species (ROS) can alter the structure of DNA, as well as lead to the formation of oxidatively modified purine and pyrimidine bases, DNA strand breaks, DNA-protein cross-links, abasic sites and other types of DNA lesions [2]. The reliable measurement of oxidatively induced DNA damage *in vitro* and *in vivo* is critical to the establishment of reference ranges for biomarkers of redox imbalance in health and disease. As was recognized early-on, the overall variation in DNA lesion levels, obtained by different analytical methods may reach up to tenfold even using the same samples derived from human cells [3]. Interlaboratory comparison studies with calf thymus DNA (ct-DNA) samples containing increasing amounts of 8-hydroxyguanine (8-OH-Gua) lesions concluded that control or reference samples with 8-OH-Gua lesion levels close to those found in nature (≈ one lesion per 10^6^ DNA bases) are needed to ensure robust and reproducible measurements in human studies [4]. 8-OH-Gua is not only an important DNA damage lesion but also a recognized biomarker of oxidative stress. Lack of dependable DNA damage reference materials and standardized measurement methods, despite many case-control studies, hampers the wider recognition of the link between oxidatively damaged DNA and disease.

The effective redox potential is a specific metric that can be used to quantify the relative strength of different oxidizing agents, including different types of ROS. Hydroxyl radicals (^•^OH), although short-lived (τ≈10^-9^s) [5], are one of the strongest oxidants generated in aqueous environments with a formal potential of E_7_ = +1.9 V (Ag/AgCl, saturated KCl) [6]. Other ROS such as hydrogen peroxide (H_2_O_2_): E_7_ = + 0.6 V [6] or superoxide radical (O_2_^•−^): E_7_ = − 0.13 V [7] are less potent, but more stable in solution. Establishing the relationship between the strength/power of the exogenous oxidative action, characterized by the imposed redox potential, and the subsequent biomolecular consequences is essential for characterizing the mechanistic framework of oxidative damage [8]. Environments that induce oxidative stress in biological experiments are traditionally generated via exposure to ionizing radiation, UV light or oxygen. Exposure to chemical agents or chemical reactions that produce ROS can also produce oxidizing environments [9]. However, in many of these exposure scenarios the oxidant strength is hard to control and quantify in such environments and is typically presented in relative and/or qualitative terms that impedes data comparison across laboratories.

DNA contains four electroactive bases that can be electrochemically oxidized [10–12]. This important feature led to the development of electrochemical sensors for detecting DNA hybridization and DNA base damage at attomole levels [13]. Electrochemical oxidation is rather slow and irreversible, the rate of which depends on the apparent standard redox potential and a standard rate constant [14]. Numerous electroanalytical studies have determined redox potentials of free bases [15], nucleotides and nucleosides [7, 9] [16–18] and synthetic DNA [19]. At pH 7, free guanine (Gua) is oxidized at +0.7 V(vs Ag/AgCl), adenine (Ade) at +0.97 V, thymine (T) at +1.15 V, and cytosine (Cyt) at +1.31 V [14]. Corresponding redox potentials for nucleotides increase by ≈ 0.25 V for Gua, Ade, Thy and 0.17 V for Cyt [20], while redox potentials for DNA bases inside intact DNA are further modulated by π stacking, hydrogen bonding, chemical modification and shielding of the electroactive moieties [14]. Electrochemical studies on intact single and double-stranded DNA indicate that redox potentials increase in the order of dGua<dAde<dThy<dCyt [21–23] with dThy and dCyt oxidation current waves reportedly overlapping [20]. Electro-oxidation efficiency is highly dependent on DNA structure and percentage of Gua [19]. A compilation of the nucleotide oxidation potentials is presented in Table A in S1 Text.

The oxidation of Gua is an irreversible process that occurs in two steps. The first step requires two protons and two electrons, resulting 8-OH-Gua. Another Gua damage product is 2,6-diamino-4-hydroxy-5-formamidopyrimidine (FapyGua), which forms when the intermediate 8-hydroxy-7,8-dihydroguan-8-yl radical is reduced leading to the opening of the imidazole ring [24]. The Ade oxidation mechanism is similar, resulting in 8-oxo-7,8-dihydroadenine (8-OH-Ade) and 4,6-diamino-5-formamidopyridimidine (FapyAde) as the key stable products. Ring-opened lesions are cytotoxic as they interfere with DNA synthesis [25]. The Ade oxidation product yield is typically eight- to tenfold lower than the corresponding yields of Gua-derived products [26].

As pyrimidines possess higher oxidation potentials than purines, they would be expected to be less susceptible to oxidative damage based on thermodynamics. Nevertheless, oxidative damage in Ade/Thy rich sequences is observed mostly at Thy due to proton-coupled electron transfer assisted formation of the Thy hydroperoxyl radicals [27]. These radicals may decay via pyrimidine ring cleavage forming 5-hydroxy-5-methylhydantoin derivatives (5-OH-5-MeHyd) [26]. Accumulation of these lesions *in vivo* often indicates potential lethality as it interferes with DNA polymerase function [28].

Most electron transfers in DNA are accompanied by proton transfer, therefore nucleic acid base redox potentials are pH-dependent [29]. When an intact DNA strand is probed with electrochemical techniques, electrical charges can migrate significant distances along the strand [30, 31] facilitating the indirect electro-oxidation of nucleobases [14, 32]. Coupling of preparative electrochemical DNA oxidation with spectroscopic examination of reaction products offers new insights into these processes. Goyal et al. studied the oxidation chemistry of numerous nucleic acid components at various carbon-based electrodes and analyzed the reaction products using ultraviolet-visible (UV-VIS) spectrophotometry, nuclear magnetic resonance (NMR) spectroscopy and mass spectrometry (MS) [16, 33, 34]. Baumann et al. [35] analyzed electrochemical oxidation products of free nucleotides by using an efficient flow-through thin-layer electrochemical cell coupled to real-time product analysis via electrospray ionization mass spectrometry (ESI-MS). Their electrochemical system included a boron doped diamond (BDD) anode that could both abstract electrons directly from DNA in solution as well as produce ^•^OH radicals at high anodic potentials (E ≥ 1.8 V).

The goal of the present work was to create a stable and robust solution-state environment for electro-oxidizing genomic DNA samples using controlled potentials in a batch operating mode as a potential pathway to produce DNA damage calibrants. A BDD working electrode (WE) was utilized to establish a stable oxidizing environment, an environment whereby DNA samples with quantifiable levels of oxidatively modified lesions could be produced. Previously, with capillary electrophoresis, we have quantified nucleic acid fragmentation under a constant oxidizing potential treatment using a reticulated carbon WE [36]. Here we equilibrate solution-state DNA with a high surface area BDD WE, biased at constant potentials in the range from 0.5 V to 2.0 V. When polarized at 2V the BDD electrode becomes an efficient ^•^OH radical producer while limiting oxygen evolution due to its high overpotential [37, 38]. The maximum concentration of ^•^OH radicals at the BDD electrode surface may reach several tens of micromoles per liter and the reaction layer can extend up to a micrometer into the electrolyte [39]. Exposure of the continuously agitated DNA solution molecules to a high surface area WE battery enables a steady charge exchange between the DNA and the anodes either directly and through products of solvent electrolysis serving as redox mediators. The reverse reactions at the counter electrode (CE) are suppressed by enclosing the Si CE in dialysis bags (2000 MW cutoff) that prevent direct DNA molecule (average MW of DNA is about 800 000 [40]) access to the CE surface.

Hyphenated mass spectrometry techniques are broadly utilized for the identification and quantification of oxidatively induced DNA damage in cells, tissues and whole organisms. In the present study, ct-DNA in buffered solution was electrochemically-treated (oxidized) using a range of fixed potentials to generate controlled levels of DNA base damage (DNA lesions) within the intact, double-stranded DNA matrix. Following enzymatic release of the DNA lesions from the treated samples, isotope-dilution gas chromatography/tandem mass spectrometry (GC/MS/MS) was utilized to identify and quantify the distribution of generated DNA lesions at each BDD WE potential. DNA damage profiles generated by direct one electron oxidation at low potentials (E = 0.5 V to 1.5 V) were quantitatively compared against the DNA damage profiles produced by treating the DNA to an oxidative bias at E = 2 V, where ^•^OH radicals are known to be efficiently generated.

## Materials and methods

### Materials

Calf thymus DNA-sodium salt (ct-DNA), potassium phosphate monobasic and potassium phosphate dibasic were purchased from Sigma-Aldrich Corp (St. Louis, MO, USA). 4,6-Diamino-5-formamidopyrimidine-^13^C,^15^N_2_ (Fapy adenine-^13^C,^15^N_2_), 2,6-diamino-4-hydroxy-5-formamidopyrimidine-^13^C,^15^N_2_ (Fapy guanine-^13^C,^15^N_2_), 8-hydroxyadenine-^15^N_5_ (8-OH-adenine-^13^C,^15^N_2_), 5-hydroxy-5-methylhydantoin-^13^C,^15^N_2_ (5-OH-5-MeHyd-^13^C,^15^N_2_) and 8-hydroxy-2′-deoxyguanosine-^15^N_5_ were purchased from Cambridge Isotope Laboratories (Andover, MA). 8-Hydroxyguanine-^15^N_5_ (8-OH-guanine-^15^N_5_) was obtained by hydrolysis of 8-hydroxy-2′-deoxyguanosine-^15^N_5_ with 60% formic acid at 140°C for 30 min followed by lyophilizing. Subsequently, 8-OH-guanine-^15^N_5_ was dissolved in 10 mmol/L NaOH before use. *E*. *coli* formamidopyrimdine DNA glycosylase (Fpg) and *E*. *coli* endonuclease (III) (EndoIII) were purchased from Trevigen (Gaithersburg, MD).

### The electrochemical reactor

**Fig 1:**
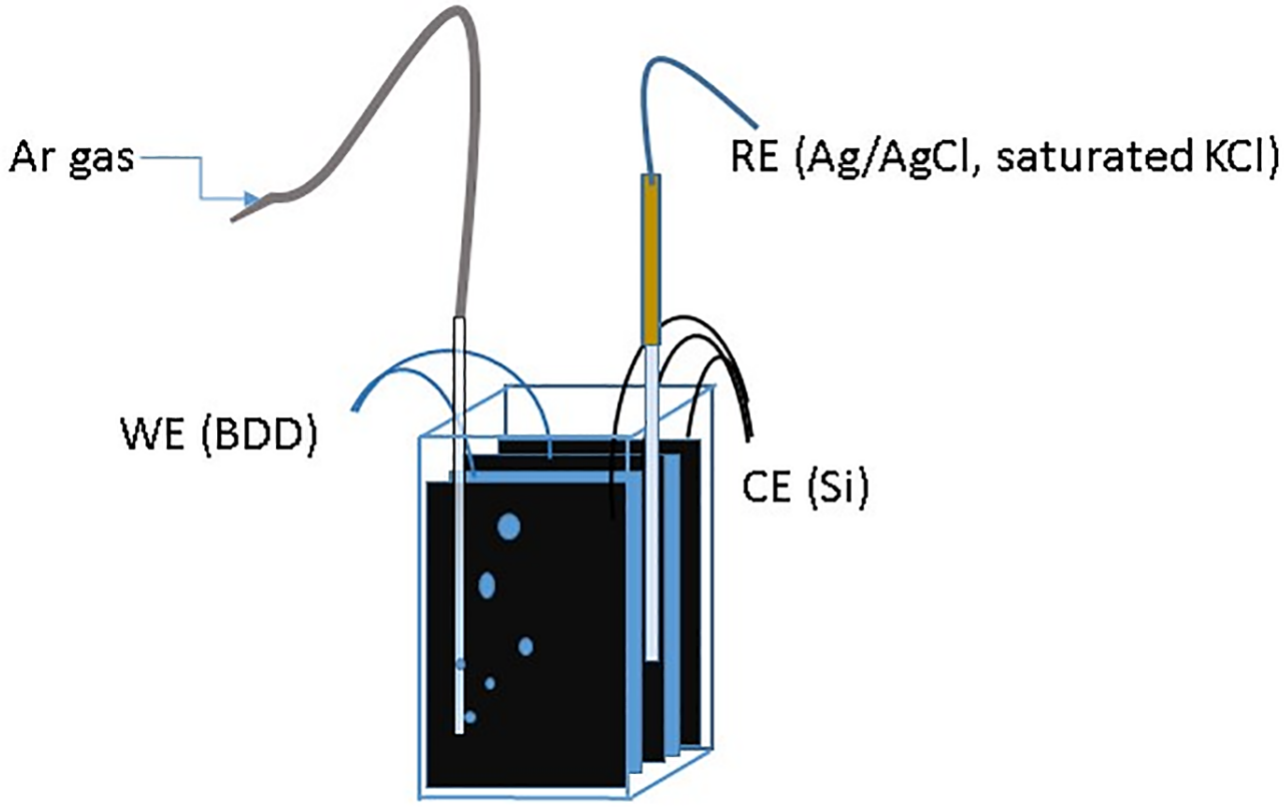
Electrochemical cell used for DNA oxidation. BDD WE is shown in blue, Si CE- black.

The stable oxidative environment was created by exposing ct-DNA in solution to the desired WE potential, maintained by an EG&G Model 273 potentiostat (Princeton Applied Research, Inc., Oak Ridge, TN)). Electrochemical cell (see Fig 1) contained a battery of two double-sided BDD WE’s (total active area S = 40 cm^2^) and three double-sided highly doped Si wafer CE’s (S = 60 cm^2^) (Virginia Semiconductor, Inc., Fredericksburg, VA), each enclosed in a 2000 MW cutoff dialysis bag (Thermo Fisher Scientific, Waltham, MA). The electrodes were separated by 0.2 mm Teflon gaskets with contact leads produced using silver epoxy. A rectangular 40 mm path-length quartz spectrophotometer cell (Starna Cells, Inc., Atascadero, CA) was used for bulk electrolysis under slow solution mixing by the inert Ar gas purging. A flexible Ag/AgCl electrode (MI-402, Microelectrodes Inc., Bedford, NH) served as a reference electrode (RE). Unless noted otherwise, the potential values throughout this study are quoted relative to the Ag/AgCl, saturated KCl RE (E = 0.199 V vs Normal Hydrogen Electrode). Prior to sample introduction, cell electrodes were activated by potential cycling at 20 mV/s in buffer solution between -1 V and +2.5 V for 30 min.

During potentiostatic treatment, the cell contained 2 mL of DNA solution in 0.01 mol/L potassium phosphate buffer (pH 7.0) at room temperature (20°C). Following each h exposure at a given WE potential, the DNA solution was removed for analysis, the cell was dismantled, and the electrodes were sonicated for 10 min in a detergent solution (RBS35, Pierce Inc., Rockford, IL), followed by a vigorous rinsing with distilled and deionized (DI) water.

### DNA sample preparation and treatment

Calf thymus DNA 0.5 mg/mL was dissolved in DI water using 50 mL Falcon tube. To fully solubilize the stock DNA, the tube was placed on a rocker for 72 h at 4°C using gentle inversion. Next, the stock solution was dialyzed against DI water using membrane dialysis with a 6 kD cutoff and stored at 4°C. The DNA concentration was measured by absorbance at 258 nm and was distributed into 50 microgram aliquots. Solutions of DNA at 250 μg/mL in 0.01 mol/L potassium phosphate buffer (pH 6.9) were prepared for each electrochemical treatment as well as controls for GC/MS/MS.

### Measurement of DNA base lesions by isotope-dilution GC/MS/MS

**Fig 2:**
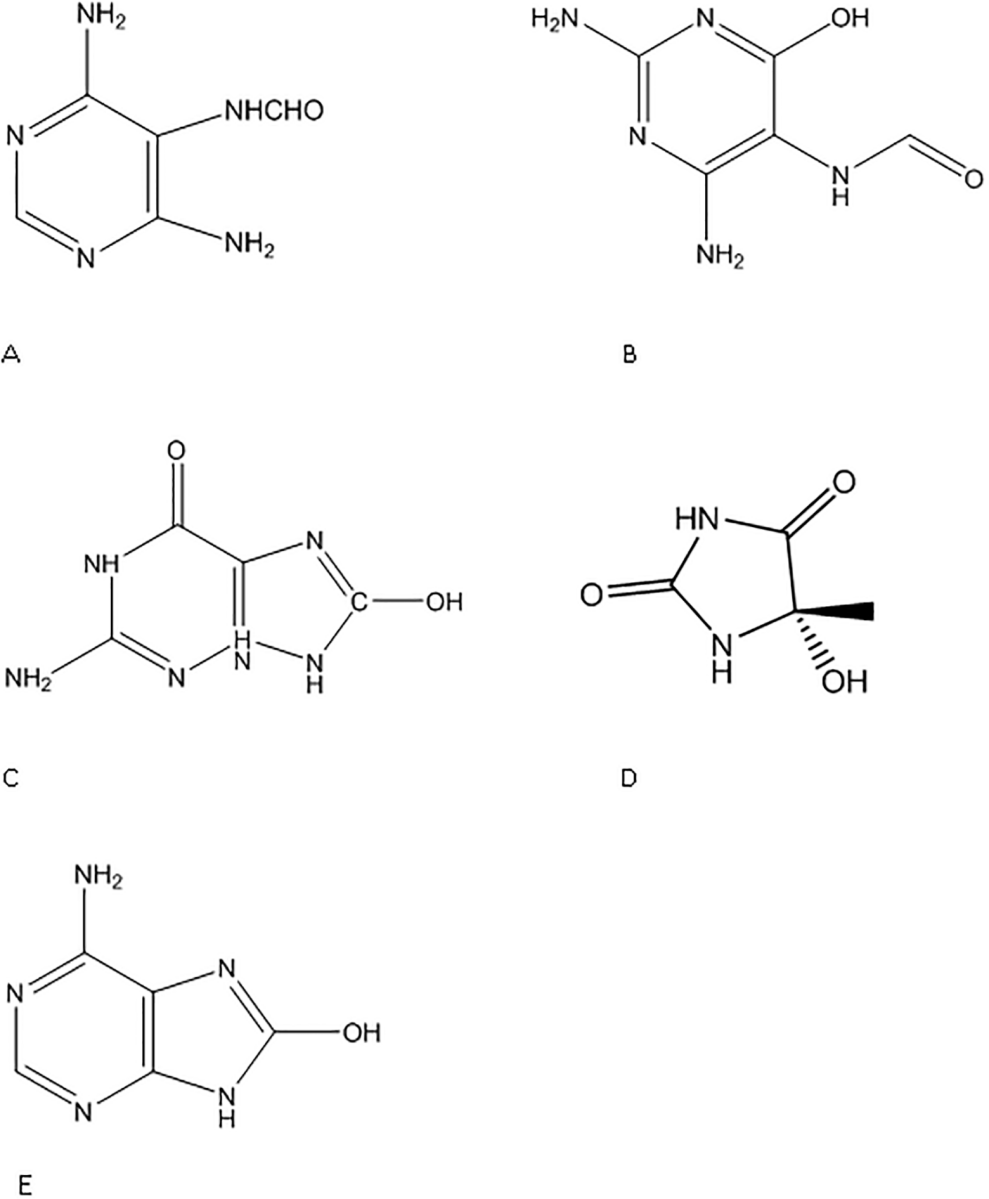
Oxidatively modified DNA base lesions measured in this study. (A) 4,6 diamino-5-formamidopyrimidine (FapyAde), (B) 2,6-diamino-4 hydroxy-5-formamidopyrimidine (FapyGua), (C) 8-hydroxyguanine (8-OH-Gua), (D) 5-hydroxy-5-methylhydantoin (5-OH-5-MeHyd), and (E) 8 -hydroxyadenine (8-OH-Ade).

Samples containing 50 μg of genomic DNA were enzymatically digested and analyzed using isotope-dilution GC/MS/MS based upon procedures described in previous studies [41–46]. The following DNA base lesions were reproducibly detected and quantified in electrochemically oxidized DNA samples: FapyAde and 8-OH-Ade (Ade-derived lesions), 5-OH-5-MeHyd (T-derived lesion) and FapyGua and 8-OH-Gua (Gua-derived lesions) (Fig 2). The reproducible detection and quantification of 5-OH-Cyt (Cyt-derived lesion) was not possible using our currently developed GC/MS/MS procedure due to interference from an overlapping component having the same retention time and mass/charge (*m/z*) ratio as 5-OH-Cyt. 40 Gy gamma irradiated Ct-DNA was used as a positive control. Full details of the methodology are available in the S1 Text.

### Statistical analysis

All statistical analyses were conducted with GraphPad Prism 6.0. Statistical tests and criteria for the reported results are given in the respective Figure legends.

## Results

The selected potential range encompassed the known oxidation potentials of the four DNA bases [23, 29, 47]. At the highest applied potential, E = 2 V, the BDD WE is also known to produce significant quantities of ^•^OH radicals through H_2_O electrolysis [38].

**Fig 3:**
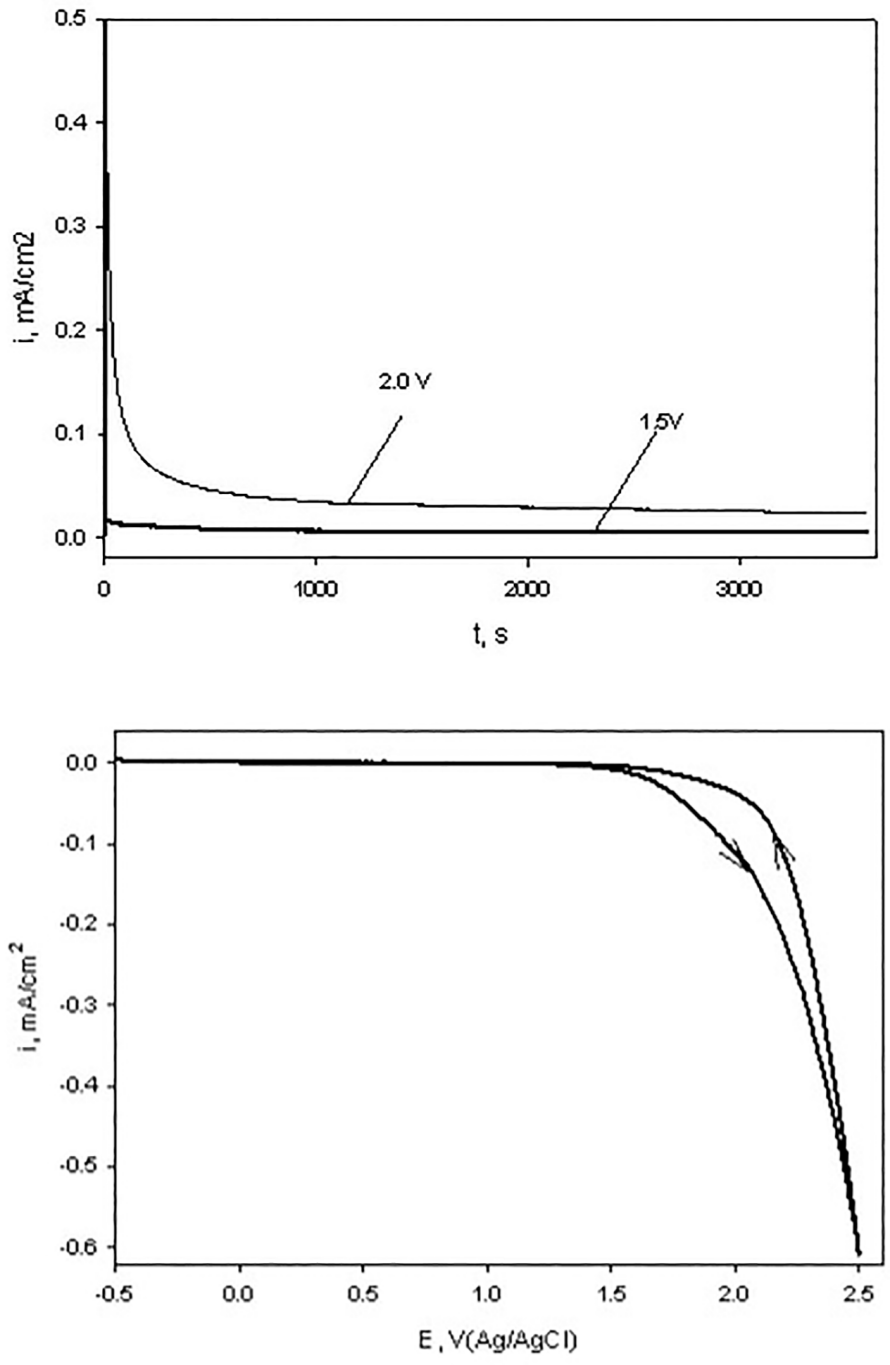
Chronoamperometry curves of the BDD electrode for two fixed potentials (upper panel) and a cyclic voltammetry trace (scan rate v = 10 mV/s, lower panel) in 0.01 mol/L potassium phosphate buffer (pH = 7) + 250 μg/mL DNA.

DNA solutions at 250 μg/mL were loaded into the electrochemical cell, slowly purged with inert gas for 30 min at the open circuit potential and a fixed potential was applied to the WE for 1 h with values: 0.5 V, 1.0 V, 1.5 V and 2.0 V. As evident from the current-time trace, recorded during 2 V potentiostatic treatment (Fig 3, upper panel), the solution species establish a redox equilibrium with WE in about 20 min. following potential application, while at lower applied potentials the cell current was very low, indicating an exhaustive DNA electrolysis. A slow linear current-potential scan, recorded in the same solution (Fig 3, lower panel) shows that the cell current starts increasing at E > 1.6 V due to the start of the H_2_O oxidation reactions at the BDD WE. As shown previously [48, 49], H_2_O oxidation on BDD electrodes proceeds via efficient generation of the ^•^OH radicals:

$$H_{2}O\to \cdot OH+H^{+}+e^{-},$$ (1)

Electrogenerated ^•^OH radicals may react with each other forming hydrogen peroxide (H_2_O_2_), which can either diffuse into the bulk of the electrolyte or oxidize to oxygen (O_2_)[48]:

$$2∙OH\to H_{2}O_{2},$$ (2)

$$H_{2}O_{2}\to O_{2}+2H^{+}+e^{-}$$ (3)

**Fig 4:**
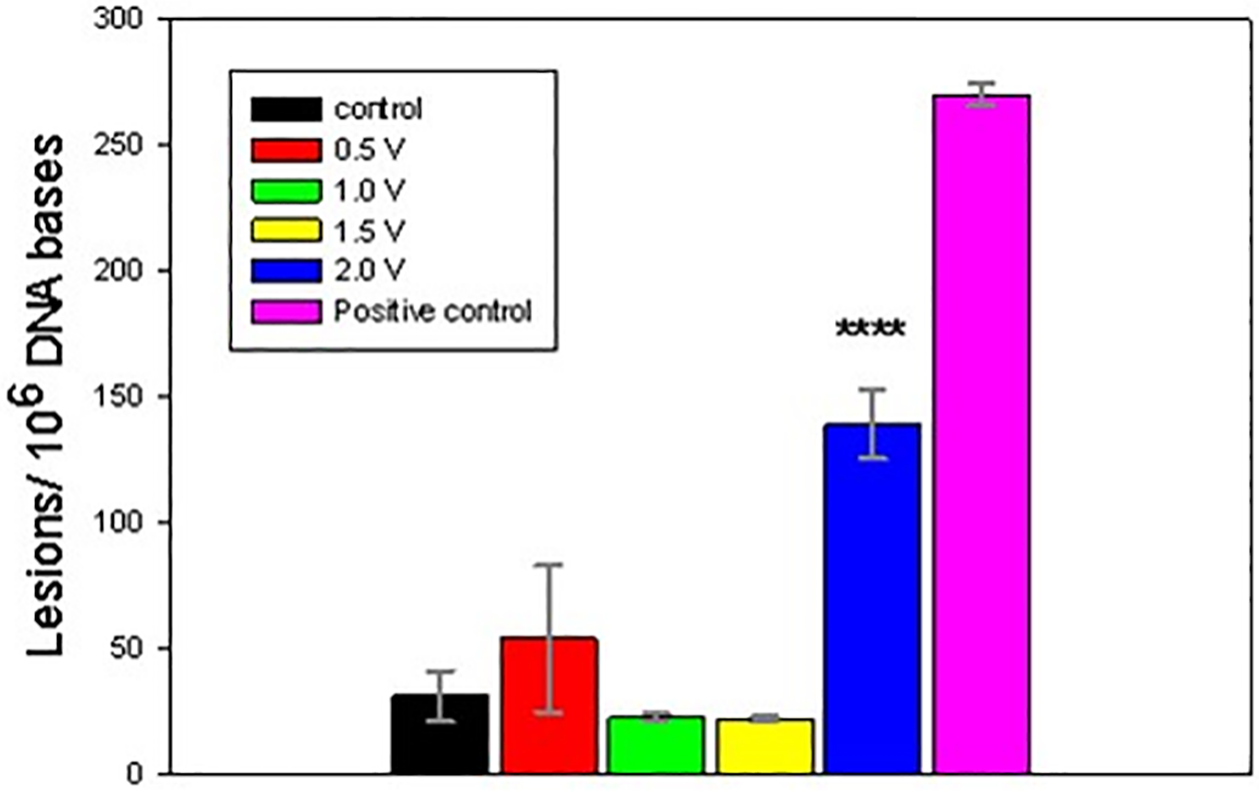
Level of 8-OH-Gua lesions, measured following 1 h DNA electrolysis at four fixed potentials. Asterisks indicate significantly increased lesion level results compared to the control samples using one-way Analysis of Variance (ANOVA) followed by Dunnett’s multiple comparison test. The analytical positive control sample was not included in the ANOVA analysis. Four asterisks indicate p < 0.0001. Control and fixed potential sample data represent the mean of 5 independent measurements. The analytical positive control data represents the mean of 2 independent measurements. Uncertainties are standard deviations.

**Fig 5:**
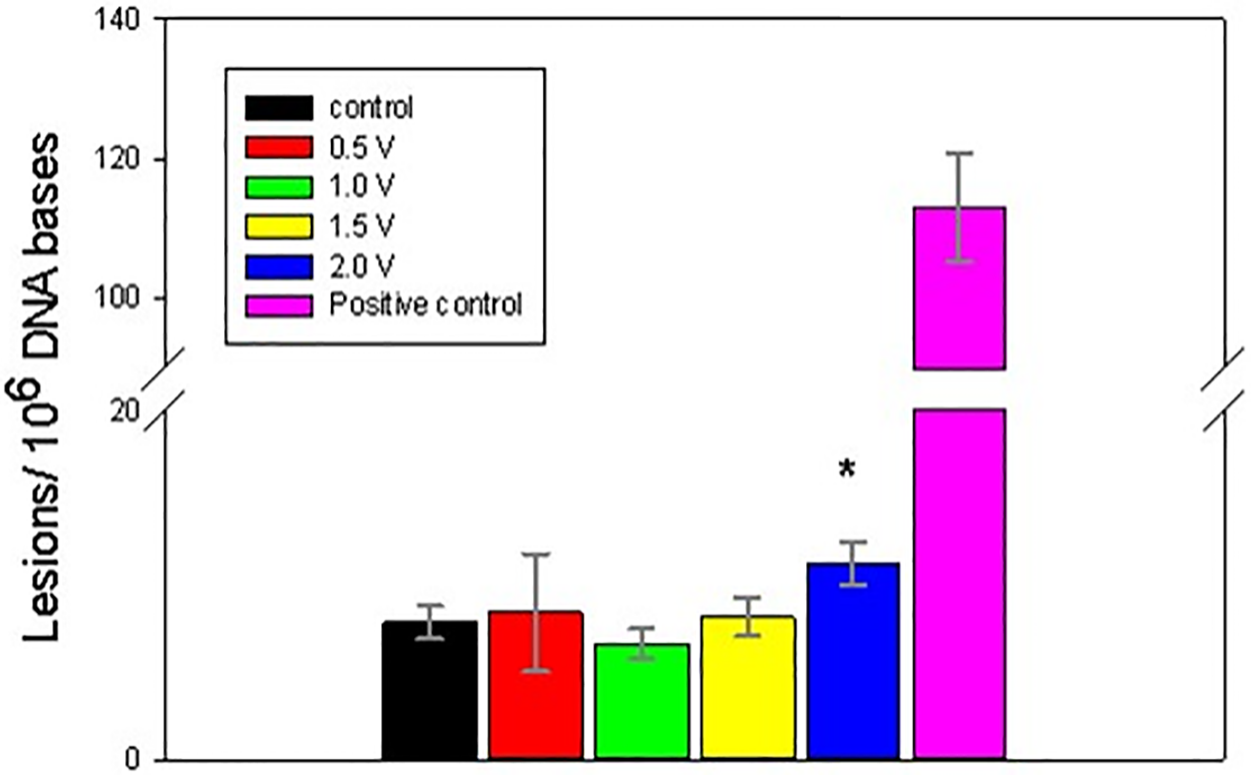
Level of FapyGua lesions, measured following 1 h DNA electrolysis at four fixed potentials. Asterisks indicate significantly increased lesion level results compared to the control samples using one-way Analysis of Variance (ANOVA) followed by Dunnett’s multiple comparison test. The analytical positive control sample was not included in the ANOVA analysis. One asterisk indicates p < 0.05. Control and fixed potential sample data represent the mean of 5 independent measurements. The analytical positive control data represents the mean of 2 independent measurements. Uncertainties are standard deviations.

**Fig 6:**
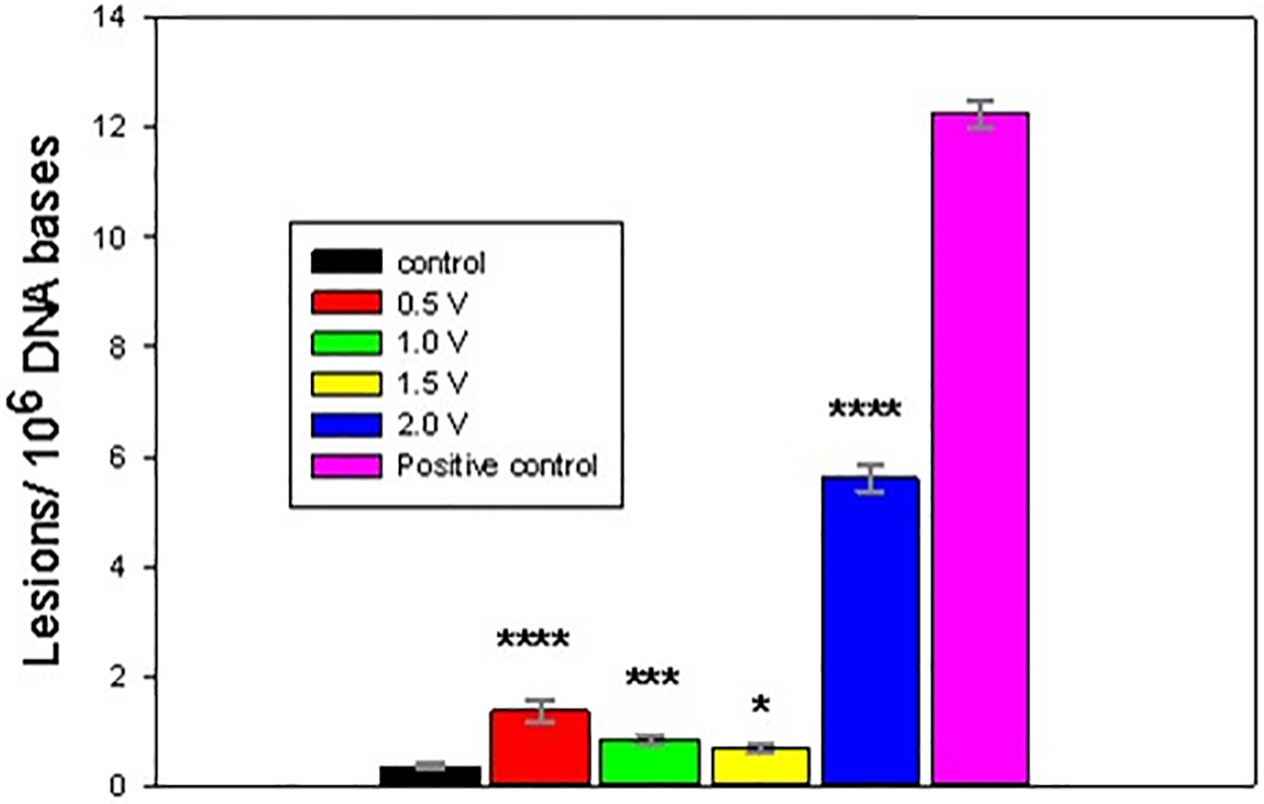
Level of 8-OH-Ade lesions, measured following 1 h DNA electrolysis at four fixed potentials. Asterisks indicate significantly increased lesion level results compared to the control samples using one-way Analysis of Variance (ANOVA) followed by Dunnett’s multiple comparison test. The analytical positive control sample was not included in the ANOVA analysis. One, three or four asterisks indicate p < 0.05, p < 0.001 or p < 0.0001, respectively. Control and fixed potential sample data represent the mean of 4 to 5 independent measurements. The analytical positive control data represents the mean of 2 independent measurements. Uncertainties are standard deviations.

**Fig 7:**
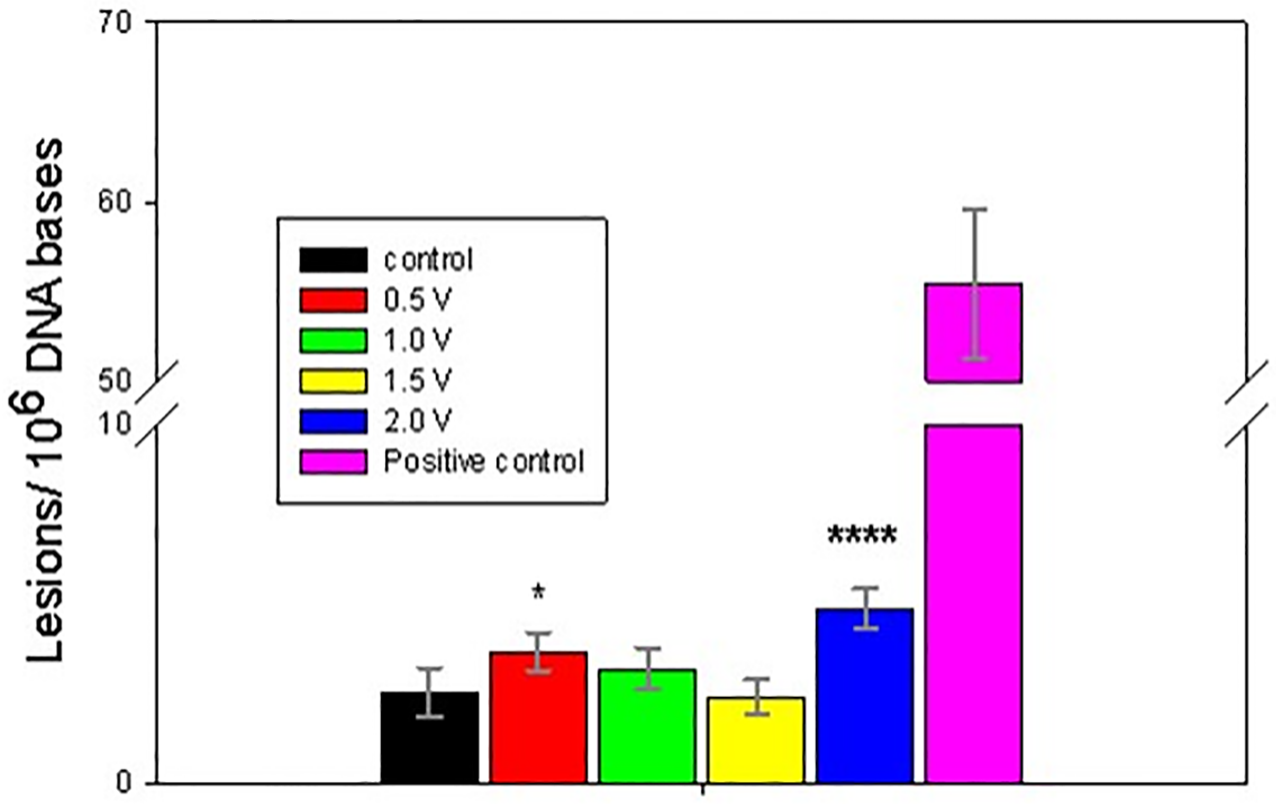
Level of FapyAde lesions, measured following 1 h DNA electrolysis at four fixed potentials. Asterisks indicate significantly increased lesion level results compared to the control samples using one-way Analysis of Variance (ANOVA) followed by Dunnett’s multiple comparison test. The analytical positive control sample was not included in the ANOVA analysis. One or four asterisks indicate p < 0.05 or p < 0.0001, respectively. Control and fixed potential sample data represent the mean of 3 to 5 independent measurements. The analytical positive control data represents the mean of 2 independent measurements. Uncertainties are standard deations.

**Fig 8:**
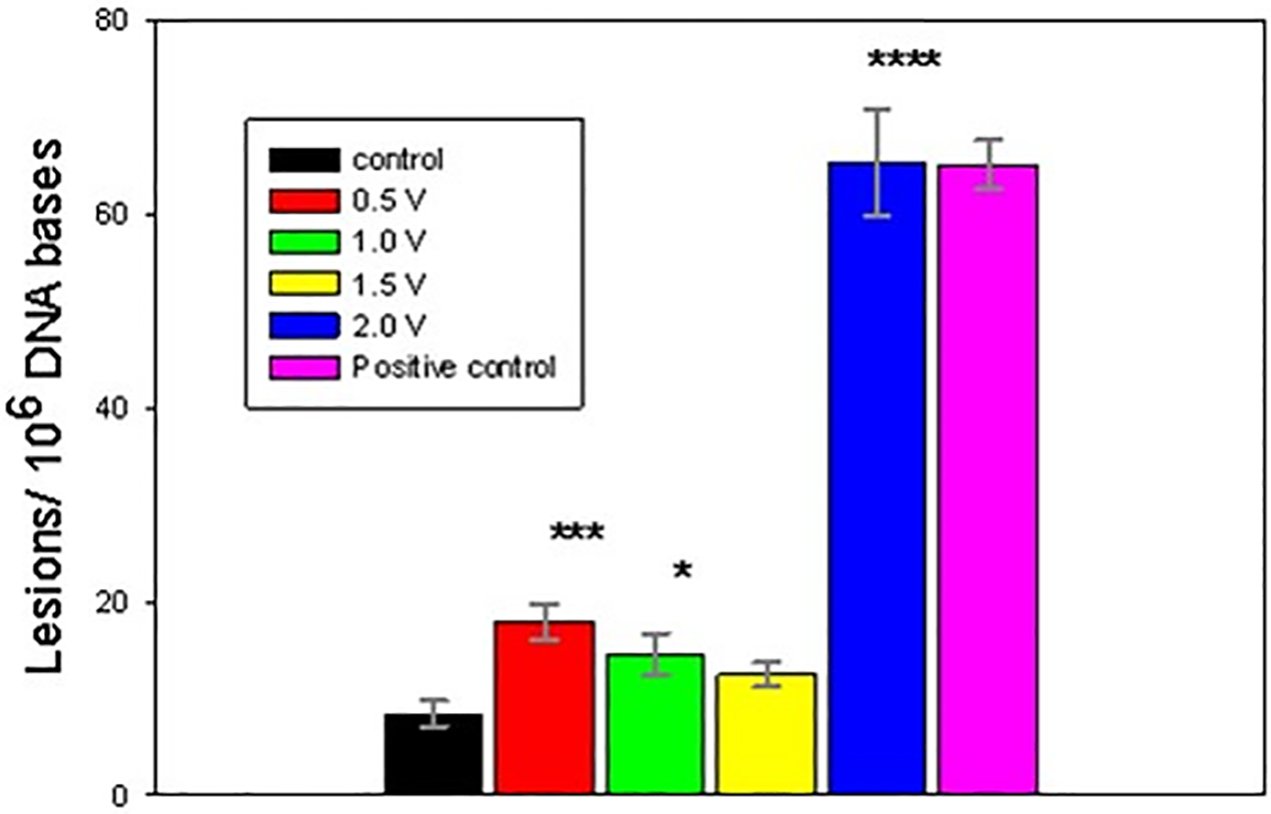
Level of 5-OH-5-MeHyd lesions, measured following 1 h DNA electrolysis at four fixed potentials. Asterisks indicate significantly increased lesion level results compared to the control samples using one-way Analysis of Variance (ANOVA) followed by Dunnett’s multiple comparison test. The analytical positive control sample was not included in the ANOVA analysis. One, three or four asterisks indicate p < 0.05, p < 0.001 or p < 0.0001, respectively. Control and fixed potential sample data represent the mean of 4 to 5 independent measurements. The analytical positive control data represents the mean of 2 independent measurements. Uncertainties are standard deviations.

The electrochemically-treated DNA was collected immediately following 1 h exposures and processed for GC/MS/MS analysis. Figs 4–8 present the measured concentrations of selected lesions produced during incubations at 4 fixed WE potential values plotted alongside the negative (DNA solution kept in the cell at the open circuit) and positive (ct-DNA treated with 40 Gy γ radiation) controls separately. Error bars correspond to one standard deviation calculated for three replicate experiments.

## Discussion

### Purine lessions

Two potential ranges could be distinguished in the oxidative lesion amount vs electrolysis potential plots: from 0.5V to 1.5V and 2V. The lower potential range includes one electron redox potentials of free purines (E_7_ G = 1.1 V, E_7_ A = 1.2 V) [15, 17, 27], while E = 2 V exceeds pyrimidine oxidation potentials (E _7_ T = 1.7V, E_7_ C = 1.6 V) [11, 29] and in addition oxidizing species, such as hydroxyl radicals, hydrogen peroxide and oxygen, which are produced by the BDD WE [38]. As evident from Figs 4–8, after electrolysis at E = 2 V a significant increase in all five measured lesion levels was observed, which happens past the onset of water electrolysis and hydroxyl radical generation at the BDD WE (Fig 3). Somewhat unexpectedly, we did not register a gradual increase in all measured lesions when DNA was exposed at increasing oxidative bias from E = 0.5 V to E = 1.5 V. Amounts of all four purine lesions were rather close to a negative control levels up to E = 1.5 V (Figs 4–7) with evidence suggesting higher levels at the lowest potential of this range (E = 0.5V). The elevated levels of 8-OH-Gua and 8-OH-Ade at E = 0.5 V could be related to a 0.5 V lower lesion oxidation potential with respect to the corresponding intact free bases (Table A in S1 Text) [18, 50–52]. Furthermore, redox potentials of purines and their oxidation intermediates in DNA are further lowered due to purine-purine stacking in the double helix as reported by Burrows et al. [53] and would favor oxidatively-induced lesion generation when equilibrated at E = 0.5V (Figs 4–7**)**. Once formed, these lesions would be vulnerable to further oxidation at increasing positive bias (when exposed at E = 1 V and E = 1.5 V), leading to the appearance of numerous other products (e.g., diamine, diol, guanidinohydantoin) [54] which were not included in the present study. *In vivo*, 8-OH-Gua and 8-OH-Ade are oxidized by tryptophyl (E = 0.9 V), tyrosyl (E = 0.73 V) and thyil (E = 0.55 V) radicals that are located in the histone proteins, covering DNA [23]. Because of its low redox potential 8-OH-Gua was also suggested to play an oxidation protective role for the DNA by serving as an “electron sink” [55].

**Fig 9:**
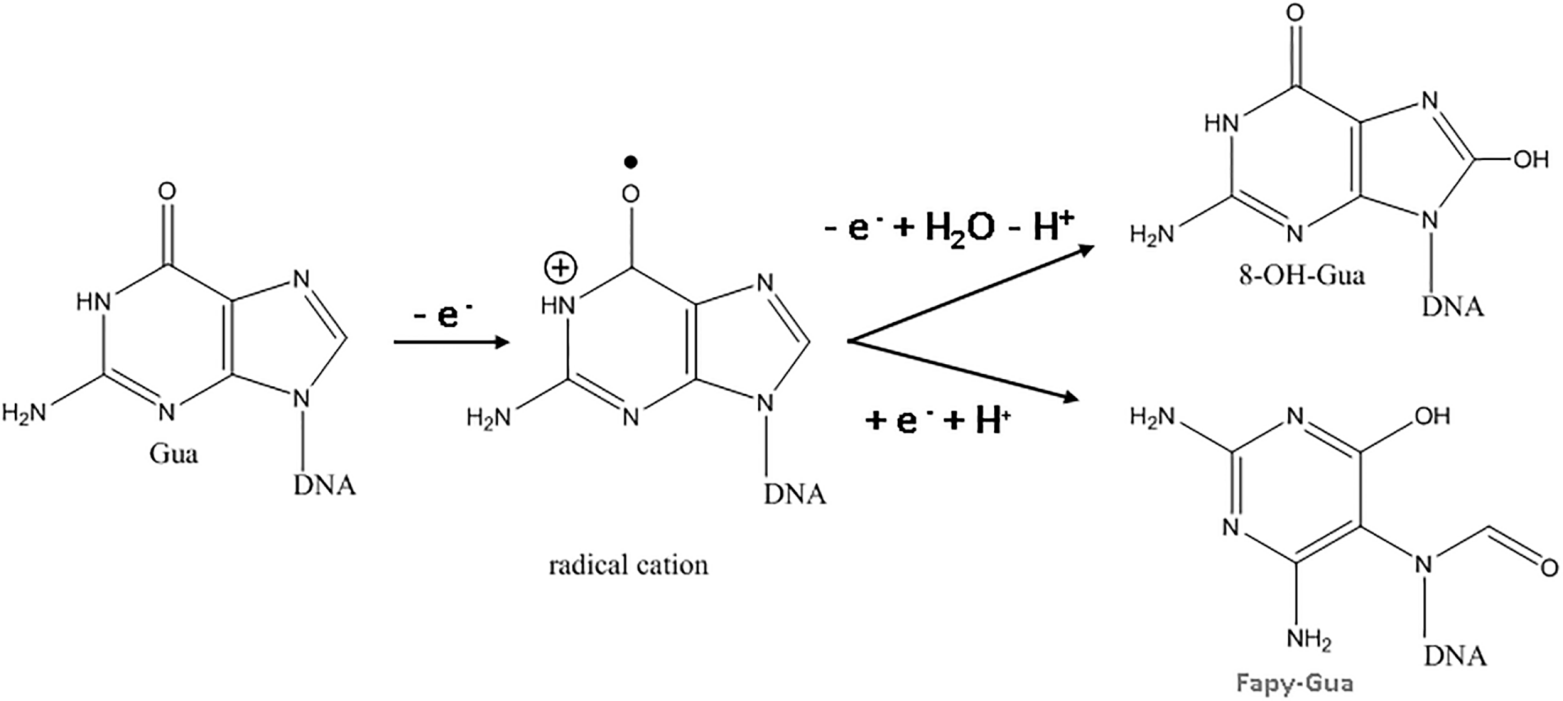
Redox ambivalence of the guanine oxidation intermediate radical cation.

The first step in the formation of 8-OH-Gua and 8-OH-Ade involves transient 8-OH-Ade adduct radical cation formation [56]. As described by Steenken et al. [57], these radicals are redox ambivalent and could be either reduced or oxidized, depending on the prevailing redox environment. The oxidative pathway will lead to the formation of 8-OH-Gua and 8-OH-Ade as final products, while the reductive pathway will result in imidazole ring-opened Fapy lesions (Fig 9, Gua only shown, Ade similar).

Both radical cation reaction pathways are consistent with the observed decrease in purine 8-OH and Fapy lesions at higher oxidative (or less reductive) potential in the range from 0.5 V to1.5 V (Figs 4–7). At 0.5 V both 8-OH-Gua and 8-OH-Ade will be exposed close to their redox equilibrium potential and the driving force for further redox conversion will be small resulting in their stabilizations at lower potentials. When the potential is shifted positive, the concentration of the detected lesions will depend on the relative rates of oxidized base formation and their transformation due to subsequent redox and/or chemical reactions. A gradual decrease in the amounts of 8-OH-Gua and 8-OH-Ade lesions from 0.5 V to 1.0 V to 1.5 V may suggest that the intermediate transformation reactions are favored at least up to 2 V, when the ^•^OH radical enabled processes of OH adduct formation and direct H abstraction overcome direct electron abstraction reactions (Figs 4–7). However, bearing in mind the experimental uncertainties, an extended DNA exposure at 0.5 V < E < 1.5 V will be required to underpin the above interpretation.

The 8-OH/Fapy lesion concentration ratio was proposed as a proxy for characterizing the *in vitro* and *in vivo* intracellular redox environment [25, 58]. Furthermore, studies by Malins et al. demonstrated that the concentration ratio of at least one ring-opened (cytotoxic) DNA base lesion to at least one hydroxyl adduct (mutagenic) DNA base lesion could be an accurate diagnostic predictor of a cancerous or non-cancerous specimen [47,48]. It is notable that in our experiments 8-OH-Gua yields were up to ten times higher than FapyGua, however the ratio was reversed for adenine lesions (Table 1). Also, both ratios approached the values in the untreated DNA sample (which was used as a negative control) and to the 40 Gy γ—irradiated positive control except for the sample oxidized at 2 V. DNA treatment at 2 V significantly enhances 8-OH lesion production over their Fapy counterparts (Table 1). Apparently, the continuous presence of the strong oxidizing environment at this potential stabilizes OH adduct lesions at the expense of ring-opened ones. Moreover, this stable and high oxidative bias boosts 8-OH-Gua yield, which is almost 30 times higher than 8-OH-Ade at 2 V. While ^•^OH radical production is likely the primary reagent in both situations, the redox environments for the bases could differ substantially. Ionizing radiation produces hydrogen atoms and hydrated electrons in addition to ^•^OH radicals, which may lower the overall redox potential, as experienced by DNA [54]. High yields of pyrimidine lesions (5-OH-5MeHyd), detected in our DNA samples exposed to 2 V (Fig 8) also suggests the role of strong oxidative bias facilitating the oxidation of OH-adduct radicals of T. Alternatively, due to its lower oxidation potential, electrons from intermediate G radicals can be transferred to A residues in close proximity thus protecting A at the expense of G [59].

**Table 1:** DNA lesion concentration ratios for ct-DNA following 1 h electrochemical oxidation at four fixed potentials and 40 Gy γ—irradiation. Values are mean and one standard deviation (n = 3) on three replicates.

Lesion ratio	Negative control, open circuit	E = 0.5V	E = 1.0V	E = 1.5V	E = 2.0V
**8-OH-Gua/8-OH-Ade**	**12.2 +-2.6**	**14.6+-4.9**	**7.1+-1.3**	**9.1+-2.1**	**28.5+-2.3**
**FapyGua/FapyAde**	**21.8+-3.9**	**6.2+-1.8**	**7.8+-1.1**	**12.2+-3.8**	**2.0+-0.3**
**8-OH-Gua/FapyGua**	**4.0+-1.2**	**6.4+-2.4**	**3.4+-1.3**	**2.7+-1.2**	**12.4+-1.7**
**8-OH-Ade/FapyAde**	**0.1+-0.03**	**0.4+-0.09**	**0.3+-1.3**	**0.3+-0.03**	**1.2+-0.3**
**8-OH-Gua/5-OH-5-MeHyd**	**3.7+-0.5**	**3.0+-0.7**	**1.6+-0.5**	**1.6+-0.5**	**2.1+-0.5**

### Pyrimidine lesions

The initial step in pyrimidine oxidation by direct electron transfer reactions includes efficient formation of radical cations [54, 60] that afterwards transform to stable products through hydration and deprotonation. Hydration leads to OH substituted radicals that are similar in activity to ^•^OH radicals [60, 61] while deprotonation leads to uracil. Considering that for pyrimidine bases the redox potentials are higher than for purine (E_7_Cyt = 1.6 V, E_7_Thy = 1.7 V) [47, 62] direct electron transfer related Thy lesion formation at 2 V would be expected to be slower on thermodynamic basis. It is also possible that pyrimidine radicals react with surrounding purines in a double stranded DNA, and produce tandem lesions [54, 63].

One electron oxidation reaction of Thy produces radical cations that decomposes primarily via hydration reaction, leading to formation of 5-OH-5MeHyd after the addition of oxygen and reduction [64]. It is notable that the Thy lesion level in DNA was significantly higher compared to the Ade lesion levels despite its higher redox potential (Figs 6–8). Similar effects were reported when DNA was oxidized by photosensitization with menadione [65]. A preferential Thy reactivity was related by Joseph et al. to a proton coupled inter-strand electron transfer from the oxidized Thy to Ade due to reactive intermediates in Ade/Thy rich DNA sections [27].

#### Relationship to the ionizing radiation induced DNA damage

Two main mechanisms are responsible for the formation of radiation induced DNA damage in aqueous environments: first—direct DNA base ionization or electron abstraction due to high energy photon absorption and second—formation of ROS (e.g., ^•^OH radicals) via radiolysis of water, which subsequently can attack DNA as previously described [63]. Both mechanisms, however, may lead to similar types of stable purine and pyrimidine decomposition products. In the case of direct electron abstraction, a cation radical is produced which then could undergo deprotonation or hydration. Interaction with the ^•^OH radical due to water radiolysis results in either nucleobase OH adduct formation or H atom loss, both pathways creating intermediate radicals [54]. A similar model is conceivable within an electrochemical system: direct electron abstraction from nucleotides exposed to the electrodes and reactions with ROS, generated at the BDD electrode due to water electrolysis (Eqs (1)–(3) above).

**Fig 10:**
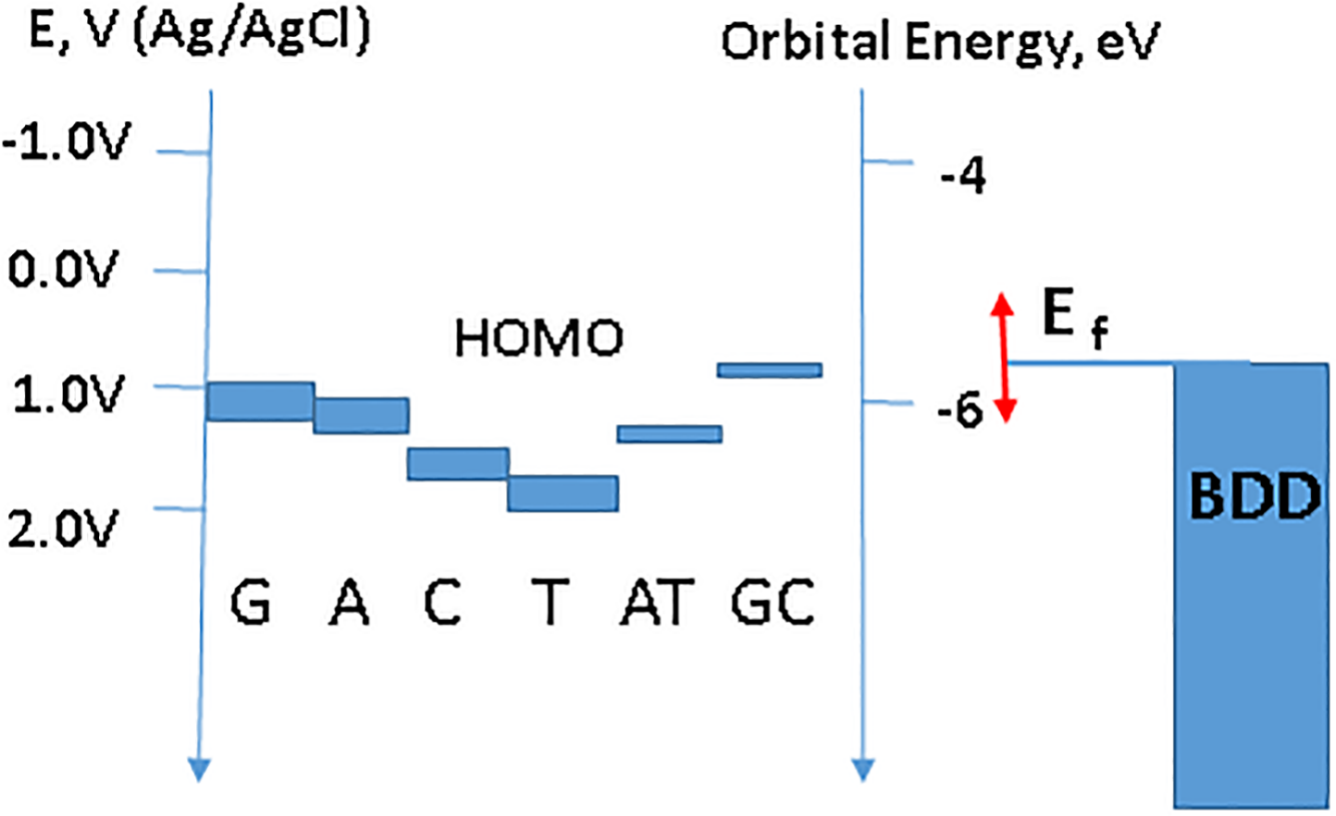
Schematic representation of the nucleic acid base highest occupied molecular orbital (HOMO) in aqueous solutions relative to the electrochemical potential scale. HOMO values are from [65–68] with bar widths representing a spread in literature values. BDD shows a continuum of the anode electronic density, red arrow—Fermi level variation with applied potential (see text).

In this context it is instructive to relate electrochemically formed lesions following DNA exposure at increasing oxidation potential with DNA damage products resulting from its exposure to photons with energies close to the DNA base ionization threshold [66, 67]. Equilibration of the DNA with a BDD electrode at a potential exceeding specific nucleobase oxidation potential facilitates one electron oxidation and is analogous to its exposure to the radiation with a photon energy exceeding base electron affinity. A sub-ionization energy photons causes direct electron excitation from HUMO and leads to cyclobutene pyrimidine dimer formation [67]. Therefore, base electron abstraction by the WE mechanistically is comparable to a direct ionization via excitation across the HUMO-LUMO (lowest unoccupied molecular orbital) gap as indicated by the UV light absorbance around 260 nm (also known as X band) [68]. Fig 10 displays nucleobase and base-pair highest occupied molecular orbital (HOMO) energy levels estimated in [69–71] relative to vacuum electron energy (right axis) and on a Ag/AgCl potential scale (left axis). Given the assumptions, used in these calculations, as well as covalent and non-covalent interactions in a double helix, these values are to be treated as approximate, nevertheless the relative nucleic acid base oxidation potentials were experimentally confirmed [14, 20, 32]. As the BDD electrode potential increases, its Fermi level E_f_ (located at the top of the filled electron energy band for a semi-metal BDD) moves down on an absolute energy scale and allows for a direct charge injection from the HOMO’s of nucleobases upon crossing it similar to ionization by a photon when its energy exceeds nucleobase electron affinity. The efficient charge transport through DNA would facilitate redox equilibration with nucleotides that may not be in direct contact with the electrode surface [72, 73].

In the present work, the goal was to quantify the extent of DNA damage induced by exhaustive electro-oxidation under well controlled conditions that mimic the effects of oxidative stress, caused by oxygen metabolism, chemical toxicants and ionizing radiation. We have shown that equilibrating soluble nucleic acids with a high surface area BDD WE maintained at a constant potential leads to oxidatively induced DNA damage. Five DNA lesions were identified and measured by GC/MS/MS: FapyAde, FapyGua, 8-OH-Gua, 5-OH-5-MeHyd and 8-OH-Ade. There were two base lesion formation modes detected in the explored electrode potential range, corresponding to 0.5 V < E < 1.5 V and E > 1.5 V. Amounts of all four purine lesions were rather close to a negative control levels up to E = 1.5 V (Figs 4–7) with evidence suggesting higher levels at the lowest potential of this range (E = 0.5 V). A statistically significant gradual drop in some measured lesions (8-OH-Ade; FapyAde, and 5-OH-5-MeHyd; Figs 6–8) was detected when DNA was exposed at increasingly higher fixed BDD WE potentials from E = 0.5 V to E = 1.5 V. We hypothesize that it could be related to the lower redox potentials of the base OH adduct lesions and redox ambivalence of the base radical cations. A substantial rise in all base lesion yields was measured when DNA was exposed at E = 2 V, when ^•^OH radicals are known to be efficiently produced by the BDD electrode. We recorded an almost 30 times higher 8-OH-Gua yield than 8-OH-Ade at E = 2 V, which also surpasses a similar ratio for a 40 Gy γ -irradiated sample. Taken together with the evidence of the elevated yields of pyrimidine lesions at this potential it shows that a stable redox bias in an electrochemical cell facilitates further base OH-adduct radical oxidation.

## Conclusions

Exhaustive DNA electrooxidation at tightly controlled potentiostatic conditions allows for the selective generation of oxidatively modified DNA lesions by either direct electron transfer or by ^•^OH radical reactions. A 1 h exposure of solution-state ct-DNA to a BDD WE, biased from 0.5 V to 2 V (vs Ag/AgCl) offers a viable method for generating an oxidative environment without the need for chemical oxidants. This experimental approach may be helpful for the development of DNA damage reference materials, a critical measurement element in establishing a quantitative link between DNA degradation and disease.

## NIST Disclaimer

Certain commercial equipment, instruments and materials are identified in this paper to specify an experimental procedure as completely as possible. In no case does the identification of particular equipment or materials imply a recommendation or endorsement by the National Institute of Standards and Technology nor does it imply that the materials, instruments, or equipment are necessarily the best available for the purpose.

## Supporting information

S1 Text Detailed methodology for GC/MS/MS determination of DNA base lesion profiles. (DOCX)
