# Supplementary material for: Controlled potential electro-oxidation of genomic DNA
Source: PLoS One. 2018 Jan 11;13(1):e0190907. doi: 10.1371/journal.pone.0190907 (PMC5764341; doi:10.1371/journal.pone.0190907)
Supplement: S1 Text — (DOCX) [file pone.0190907.s001.docx]

**S1 Text.** **Detailed methodology for GC/MS/MS determination of DNA base lesion profiles**

**Vytas Reipa****^1^, Donald H. Atha^1^, Sanem H. Coskun^1^, Christopher M. Sims^1^, Bryant C. Nelson^1^**

National Institute of Standards and Technology, Biosystems and Biomaterials Division, Materials Measurement Laboratory, Gaithersburg, MD 20899.

To prepare the DNA for enzymatic digestion, 50 µg DNA samples were dissolved in a buffer consisting of 50 mmol/L sodium phosphate, 100 mmol/L potassium chloride, 1 mmol/L EDTA and 100 µmol/L dithiothreitol (pH 7.4).  To this solution, 2 µg each of *E. coli* Fpg and EndoIII were added and each sample was digested at 37 °C for 1 h. The digestion was terminated with the addition of ice-cold absolute ethanol in combination with sample storage at – 20 °C.

Samples were centrifuged at 14000 gn for 30 min, supernatant fractions containing the excised DNA lesions were transferred to glass vials and the solvent was evaporated under vacuum.  Samples were solubilized in nuclease-free water, lyophilized, and then trimethylsilylated using bis(trimethylsilyl)trifluoroacetamide)/1 % trimethylchlorosilane in pyridine (120 °C for 30 min).  Following derivatization, samples were analyzed by GC/MS/MS.

Oxidatively modified DNA base lesions were identified and quantified using GC-MS/MS with isotope-dilution in the multiple reaction monitoring (MRM) mode. This analysis was conducted based on modifications to a previously developed selected ion monitoring (SIM) mode gas chromatography/mass spectrometry (GC/MS) methodology. Mass spectrometry analyses were performed on an Agilent 7000 series triple quadrupole GC/MS/MS system (Agilent Technologies, Santa Clara, CA) operated in positive ion mode with electron ionization. The modular system consisted of a 7693 autosampler, a 7890A GC oven and a 7000 series triple quadrupole mass analyzer set to widest resolution for MS1 and MS2.

In the current MRM mode method, specific reaction transitions for five lesions, namely 4,6-diamino-5-formamido-pyrimidine (FapyAde), 2,6-diamino-4-hydroxy-5-formamidopyrimidine (FapyGua), 8-hydroxyadenine (8-OH-Ade), 5-hydroxy-5-methylhydantoin (5-OH-5-MeHyd) and 8-hydroxyguanine (8-OH-Gua), as well as for their stable isotopically-labeled analogues (Fapy adenine-^13^C,^15^N_2_, Fapy guanine-^13^C,^15^N_2_, 8-OH-adenine-^13^C,^15^N_2_, 5-OH-5-MeHyd-^13^C,^15^N_2_ and 8-OH-guanine-^15^N_5_) were identified and optimized on the basis of the original SIM ions. The isotopically-labeled lesion analogues function as internal standards (ISTDs) for lesion quantification. The relevant MRM mass transitions were: *m*/*z* 369 → *m*/*z* 354 and *m*/*z* 372 → *m*/*z* 357.1 for FapyAde and FapyAde-^13^C,^15^N_2_ respectively; *m*/*z* 457 → *m*/*z* 442 and *m*/*z* 460 → *m*/*z* 445 for FapyGua and FapyGua-^13^C,^15^N_2_, respectively; *m*/*z* 367 → *m*/*z* 352 and *m*/*z* 370 → *m*/*z* 355 for 8-OH-Ade and 8-OH-Ade-^13^C,^15^N_2_ respectively; *m*/*z* 331 → *m*/*z* 331 and *m*/*z* 334 → *m*/*z* 334 for 5-OH-5-MeHyd and 5-OH-5-MeHyd-^13^C,^15^N_2_ respectively and *m*/*z* 455 → *m*/*z* 440 and *m*/*z* 460 → *m*/*z* 445 for 8-OH-Gua and 8-OH-Gua-^15^N_5_, respectively. Final results are reported in terms of the number of lesions quantified / 10^6^ DNA bases.

**Table A. Oxidation potentials of nucleotides**

| Nucleotide | E, V (Ag/AgCl) at pH = 7 | reference |
| --- | --- | --- |
| Gua (in ssDNA) | 0.9 | [1] |
| Gua (guanosine radical) | 1.09 | [2] |
| Gua (free base) | 0.7 | [3] |
| 8-OH-Gua | 0.4 | [4] |
| 8-OH-Gua | 0.54 | [5] |
| 8-OH-Gua | 0.50 | [6] |
| A (AMP) | 1.26 | [7] |
| Ade(free base) | 1.34 | [7] |
| Ade(adenosine radical) | 1.22 | [2] |
| 8-OH-Ade | 0.55 | [8] |
| Ade(mononucleotide) | 1.23 | [3] |
| Thy (TMP) | 1.51 | [7] |
| Thy(free base) | 1.96 | [7] |
| Cyt(free base) | 1.77 | [7] |
| Cyt(CMP) | 1.89 | [7] |

1. Oliveira-Brett AM, Piedade JAP, Silva LA, Diculescu VC. Voltammetric determination of all DNA nucleotides. Analytical biochemistry. 2004;332(2):321-9. doi: 10.1016/j.ab.2004.06.021. PubMed PMID: WOS:000223604100015.

2. Steenken S, Jovanovic SV. How easily oxidizable is DNA? One-electron reduction potentials of adenosine and guanosine radicals in aqueous solution. J Am Chem Soc. 1997;119(3):617-8. doi: DOI 10.1021/ja962255b. PubMed PMID: WOS:A1997WD86700024.

3. Boussicault F, Robert M. Electron transfer in DNA and in DNA-related biological processes. Electrochemical insights. Chem Rev. 2008;108(7):2622-45. doi: 10.1021/cr0680787. PubMed PMID: WOS:000257666100016.

4. Brett CMA, Brett AMO, Serrano SHP. On the Adsorption and Electrochemical Oxidation of DNA at Glassy-Carbon Electrodes. J Electroanal Chem. 1994;366(1-2):225-31. doi: Doi 10.1016/0022-0728(93)02994-S. PubMed PMID: WOS:A1994NC47200024.

5. Steenken S, Jovanovic SV, Bietti M, Bernhard K. The trap depth (in DNA) of 8-oxo-7,8-dihydro-2 ' deoxyguanosine as derived from electron-transfer equilibria in aqueous solution. J Am Chem Soc. 2000;122(10):2373-4. doi: DOI 10.1021/ja993508e. PubMed PMID: WOS:000086050900029.

6. Goyal RN, Jain N, Garg DK. Electrochemical and enzymic oxidation of guanosine and 8-hydroxyguanosine and the effects of oxidation products in mice. Bioelectroch Bioener. 1997;43(1):105-14. doi: Doi 10.1016/S0302-4598(96)05182-3. PubMed PMID: WOS:A1997XW73000017.

7. Brotons A, Vidal-Iglesias FJ, Solla-Gullon J, Iniesta J. Carbon materials for the electrooxidation of nucleobases, nucleosides and nucleotides toward cytosine methylation detection: a review. Anal Methods-Uk. 2016;8(4):702-15. doi: 10.1039/c5ay02616d. PubMed PMID: WOS:000368943700001.

8. Wagner JR, Hu CC, Ames BN. Endogenous Oxidative Damage of Deoxycytidine in DNA. Proceedings of the National Academy of Sciences of the United States of America. 1992;89(8):3380-4. doi: DOI 10.1073/pnas.89.8.3380. PubMed PMID: WOS:A1992HP04300046.

**NIST Disclaimer**

Certain commercial equipment, instruments and materials are identified in this paper to specify an experimental procedure as completely as possible. In no case does the identification of particular equipment or materials imply a recommendation or endorsement by the National Institute of Standards and Technology nor does it imply that the materials, instruments, or equipment are necessarily the best available for the purpose.
